# Preliminary Studies on Biodegradable Zinc Oxide Nanoparticles Doped with Fe as a Potential Form of Iron Delivery to the Living Organism

**DOI:** 10.1186/s11671-019-3217-2

**Published:** 2019-12-10

**Authors:** Paula Kielbik, Jarosław Kaszewski, Bartłomiej Dominiak, Magdalena Damentko, Izabela Serafińska, Julita Rosowska, Mikołaj A. Gralak, Marcin Krajewski, Bartłomiej S. Witkowski, Zdzislaw Gajewski, Marek Godlewski, Michal M. Godlewski

**Affiliations:** 1 Department of Physiological Sciences, Faculty of Veterinary Medicine, Warsaw University of Life Sciences, Nowoursynowska 159, 02-776 Warsaw, Poland; 2 Veterinary Research Centre, Centre for Biomedical Research, Department of Large Animal Diseases with Clinic, Faculty of Veterinary Medicine, Warsaw University of Life Sciences, Nowoursynowska 100, 02-797 Warsaw, Poland; 3 Institute of Physics, Polish Academy of Sciences, Al. Lotnikow 32/46, 02-668 Warsaw, Poland; 4 Department of Preclinical Sciences, Faculty of Veterinary Medicine, Warsaw University of Life Sciences, Ciszewskiego 8, 02-786 Warsaw, Poland; 5 Institute of Fundamental Technological Research, Polish Academy of Sciences, Pawińskiego 5B, 02-106 Warsaw, Poland

**Keywords:** ZnO:Fe, Nanoparticles, Iron deficiency, Iron delivery, Iron doping

## Abstract

Iron is the crucial element for living organisms and its deficiency is described as the most common nutritional disorder all over the world. Nowadays, more effective and safe iron supplementation strategies for both humans and animals become one of the most important challenges in the therapy of nutritional deficiencies. Our previous in vivo studies confirmed safety and biodegradability of in-house manufactured zinc oxide-based nanoparticles and their rapid distribution to majority of organs and tissues in the body. In vitro examinations performed on Caco-2 cell line, a model of epithelial cells of the gastrointestinal tract, revealed a low toxicity of studied nanomaterials. In the current study, we investigated biodegradable zinc oxide nanoparticles doped with Fe(III) as a perspective supplementation strategy for iron deficiency. Biodegradable ZnO:Fe nanoparticles were intra-gastrically administered to adult mice and following 24 h, animals were sacrificed with collection of internal organs for further analyses. The iron concentration measured with atomic absorption spectrometry and histological staining (Perl’s method) showed a rapid distribution of iron-doped nanoparticles to tissues specifically related with iron homeostasis. Accumulation of iron was also visible within hepatocytes and around blood vessels within the spleen, which might indicate the transfer of Fe-doped nanoparticles from the bloodstream into the tissue. Reassuming, preliminary results obtained in the current study suggest that biodegradable ZnO nanoparticles doped with Fe might be a good carriers of exogenous iron in the living body. Therefore, subsequent investigations focus on determination an exact mechanisms related with an iron deposition in the tissue and influence of nanoparticle carriers on iron metabolism are required.

## Introduction

Iron is one of the most important component building living organisms on the Earth, including human population. The importance of this element is related with an essential role of iron in many structures and processes taking place in the body, e.g. carriage of oxygen around the whole body tissues and cells (part of hemoglobin and myoglobin), production of energy (component of mitochondrial respiratory chain proteins within) any many more [1, 2]. Thus, iron takes part in crucial life processes of cells and entire organism and its deficiency may leads to significant disturbances in the functioning of the living body.

As worldwide reports show the iron deficiency becomes serious public health problem in human population. According to WHO, nearly 30% of the society all over the world was affected by anemia in years 1993–2005. Prevalence of anemia is still much greater in young children and pregnant women [3]. The importance of iron supplementation in pregnancy is related with fact that iron deficiency is a risk factor for preterm delivery, low birth weight of neonates, and their general worse health condition [4]. In case of infants and young, pre-school children, the risk factor of iron deficiency is caused by rapid growth of the whole body and subsequent consumption of the iron stores; thus, iron supplementation is also recommended for children between 0 and 5 years old [5]. Interestingly, there are scientific reports indicating the correlation of proper iron level in children and their emotional and neuropsychological development [6]. Moreover, iron deficiency is also a serious nutritional disorder among other mammalian species, especially in suckling piglets, where limited intrauterine supply of this element is coupled with low iron content in sow milk [7, 8]. Also, a rapid growth of the piglets quickly depletes remains of iron stores, as within a few days newborn piglets double their body weight, thus increasing the number of red blood cells and general blood volume. Thus, nowadays more effective and safe iron supplementation strategy for both human and animal population became one of the most important challenges in the prevention and therapy of nutritional deficiencies.

There are three major ways of an extra iron delivery to the living organism: intravenous, intramuscular, and oral. Each of them demonstrates both advantages and disadvantages of the method. Oral administration seems to be simple and more natural for the body, involving physiological pathways of iron absorption and transport systems. Also, this method preserve a natural system controlling the amount of circulating iron in the body based on hepcidine [9]. However, oral route of supplementation is often plagued with poor absorption and final low efficiency. Scientific studies reported also that the standard, soluble oral iron supplementation strategy strongly affects the composition and metabolic activity of animal gut microbiome [10]. On the other hand, intravenous or intramuscular ways of iron delivery to the organism are hampered by risk of toxic side effects [11]. Additionally, in industrial pig farming, the standard method of iron supplementation based on individual, intramuscular administration of single dose of iron-dextran is stressful for animals and troublesome for breeders. Also, improperly performed injection can cause inflammation and other complications in animals, as well as contribute to spreading various diseases in the herd, when sterility conditions are not observed.

Mentioned above toxic side effects of exogenous iron delivery to the living organism are associated with occurrence of free and unbound iron element in the body, which as a transition metal (through change in valence) can lead to production of harmful free radicals (namely reactive oxygen species—ROS) through Fenton reaction [12]. Scientific reports indicated a risk of increased level of free radicals in the organism as a factor contributing to various conditions including: bacterial infections, neoplasia, or liver diseases [13]. Natural, physiological strategy of preventing the generation of ROS by free iron is based on bounding iron element with particular proteins at each step of its absorption and transport pathways within the body. In case of oral iron overload and its intravenous/intramuscular administration, the physiological pathways of iron circulation are generally overwhelmed resulting in rapid increase of ROS [14].

Iron-based nanoparticles (Fe-based NPs) have been widely studied as a promising tool for biomedical purposes. The most extensive use of these nanostructures is related with their magnetic properties; thus, they have been mostly tested as contrast agents for clinical diagnostic techniques aimed to tumor imaging or accumulation in particular tissues [15]. Recently, nanostructures have been also studied as a novel, more effective form of delivery of bioactive substances to the organism. NP carriers may reveal considerably increased bioavailability for the living organism, comparing with substances in bulk form, which is mostly related with their smaller size [16, 17]. In one of the study, the effectiveness of NPs containing iron was compared with ferrous sulfate (standard supplement for treating anemia) in anemic rats. Basing on the blood analyses, obtained results showed an increased iron bioavailability following oral, single dose of NPs with iron in anemic animals. In case of multiple doses of nanoparticles and ferrous sulfate, obtained results were similar [18]. Another study described obtained results of Fe-based nanomaterials as an alternative iron delivery agents on humans and a rodent model. Authors particularly emphasized a superior safety of nanoFe comparing with soluble forms of iron, which resulted in lack of iron accumulation in the intestinal mucosa and promotion of beneficial microbiota [19]. Similarly promising results were obtained in the study on anemic rats treated with Fe-based NPs capped with vitamin C, which allowed for avoiding the standard route of Fe absorption from gastrointestinal track. Obtained results indicated recovery of tested animals from the anemic disease along with improving their blood parameters within short time, comparing with standard method of iron deficiency therapy [20].

## Material and Methods

### Synthesis of Nanoparticles

Zinc oxide nanoparticles doped with iron (ZnO:Fe NPs) were synthesized using microwave hydrothermal technique. Method is well established, relatively inexpensive, biofriendly and industrially scalable, capable of introduction of different foreign ions as functional dopants [21–25], into variety of oxides [26–28]. Concentration of iron in present nanoparticles was set to 5% molar. Synthesis was conducted starting from nitrates(V): Zn(NO_3_)_2_·6H_2_O (99%, Sigma–Aldrich) and Fe(NO_3_)_3_·9H_2_O (96%, Carl Roth). We have tested various suppliers and chosen the ones that offer the most uniform product which did not contain measurable levels of non-soluble leftovers. Compounds were dissolved in distilled water: 17.63 g of zinc nitrate(V) and 1.25 g of iron(III) nitrate(V). Clear solution was then alkalized using 25% aqueous ammonia solution (Carl Roth) to pH of 8. Resulting red residue was washed using Büchner funnel, with ~ 1 l of distilled water and suction filtered. Precipitate was placed in 100-ml Teflon vessel, filled with water to 80% of volume then closed in Ertec Magnum II reactor chamber. Microwave hydrothermal process was conducted at 4 MPa by 20 min. After reaction, pale red product was collected and dried overnight at 60 °C.

### Characterization of Nanoparticles

Characterization of the product was conducted using scanning electron microscopy (SEM), photoluminescence (PL) and cathodoluminescence (CL) spectroscopy, and energy dispersive elemental analysis (EDX). SEM measurements were conducted with high-resolution (1 nm) Hitachi SU-70 microscope, equipped with characteristic radiation detector and cathodoluminescence system GATAN Mono CL3 in secondary electron (SE) and transmission (TE) modes. The photoluminescence emission and excitation spectra were recorded using Horiba/Jobin-Yvon Fluorolog-3 spectrofluorimeter, equipped with a xenon lamp as an excitation source and Hamamatsu R928P photomultiplier. Dynamic light scattering (DLS) and zeta potential were measured with DelsaMax Pro particle characterization system. Thermogravimetry analysis (TGA) was conducted using NETZSCH STA 449 F1 Jupiter thermogravimeter under argon flow. SEM measurements were conducted after deposition of the nanoparticles on the Agar polycarbonate-coated 400 copper mesh. Sample was suspended in distilled water using Sonics VCX500 ultrasonic processor, and then drop of the suspension was placed on the mesh and then dried. Sample was prepared after 2-h sedimentation of heavier particles.

### In Vitro Model

For the current study, Caco-2 cell line (Caucasian colon adenocarcinoma cells) was used, as a model of epithelial cells of the gastrointestinal tract. Cell line was obtained from The European Collection of Authenticated Cell Cultures—ECACC (Sigma–Aldrich, Cat. No. 86010202). Cells were seeded at 0.5 × 10^6^ cells/ml onto 6-well or 96-well plates and maintained in Dulbecco’s Modification of Eagle’s Medium—DMEM (Gibco), supplemented with 10% fetal bovine serum—FBS (Gibco), 1% non-essential amino acids—NEAA (Gibco), 1% penicillin–streptomycin–neomycin—PSN (Gibco), and 0.2% sodium bicarbonate (Gibco) at 37 °C and 5% CO_2_. When 95–100% confluency was observed, cell culture medium was removed and suspension of ZnO:Fe NPs in fresh growth medium was added. Cells were incubated with four different concentrations of ZnO:Fe NPs (1.0; 0.1; 0.01; and 0.001 mg of ZnO:Fe NPs/ml) for 24 h at 37 °C and 5% CO_2_.

### Cell Viability Analyses

Cell viability was assessed for Caco-2 using two methods: XTT assay and Trypan blue staining. All reagents required for the XTT assay were provided from the commercial kit (Cell Proliferation Kit II, Roche). Following the incubation of cells grown on 96-well plate with different concentrations of ZnO:Fe NPs, cells were incubated with 50 μl XTT labeling mixture for 4 h at 37 °C and 5% CO_2_. Subsequently, concentration of formazan was assessed with spectrophotometric method. The absorbance was measured at 470/650 nm and results were correlated with the number of viable cells. In the following experiment, the highest absorbance result was assumed as the 100% viability of cells. Final results were calculated based on four, separate experiment replicates (*n* = 4).

In order to perform trypan blue staining, after the cells grown on 6-well plates were incubated with different concentrations of ZnO:Fe NPs, cells were collected (0.05% tripsin and 0.2% EDTA for 10 min), centrifuged and pellet was re-suspended in 1 ml growth medium. One hundred microliter of sterile 0.4% trypan blue solution was mixed with 100 μl of cell suspension. Ten microliter of sample was loaded on the counting slide and analyzed using JuLi Br Couting Software (NanoEnTek) for assessing the number of viable cells. Final results were calculated based on three, separate experiment replicates (*n* = 3).

### Animal Model

For purposes of this preliminary study, adult, male Balb-c mice (*n* = 6) were individually kept under standard and controlled living conditions (12/12-h light-dark period, 25 °C, humidity 30%) with standard food and water (provided ad libitum). All procedures were accepted by the Local Ethical Committee, agreement no. WAW2/59/2017. Following 7 days of acclimatization, a suspension of ZnO:Fe NPs in water (10 mg/ml; 0.3 ml/mouse) was administrated by oral gavage (IG) to animals from experimental group (*n* = 4) . Mice from control group received 0.3 ml drinking water by IG (*n* = 2). During the experiment, we did not notice any behavioral changes in tested animals nor any tissue abnormalities. Following 24 h, mice from both experimental and control group were sacrificed in CO_2_-O_2_ chamber (CO2 Box, Bioscape, Merazet) with subsequent collection of tissues. Half of the freshly collected samples were frozen in − 20 °C for atomic absorption spectrometry (AAS). Second half of the tissues were fixed in 4% buffered formalin and transferred to 70% ethanol (after 24 h). Subsequently, samples were dehydrated in the increasing concentrations of ethanol, embedded in paraffin (Leica TP1020, Leica EG1150) and 6-μm-thin (or 4 μm-thin for histopathological examinations) microscopic slides were prepared with microtome (Leica RM2255).

### Iron Accumulation Assessed by Perl’s Staining

Microscopy slides of spleen, liver, and brain were stained with Perl’s method (Prussian blue) for the presence of iron. Samples were de-paraffinized and re-hydrated to distilled water, then placed in a working solution with equal parts of 5% potassium ferricyanide (Sigma–Aldrich) and 5% hydrochloric acid (Sigma–Aldrich) and stained for 30 min. Subsequently, sections were rinsed in water and counter-stained with nuclear-fast red (Sigma–Aldrich) for 5 min, rinsed in water, and mounted under the coverslips with Permount (Fisher Scientific). Images of stained samples were taken using Olympus BX60 microscope and Cell^P image acquisition software. From each tested sample, 16 randomly chosen images were selected for further examinations with MicroImage v.4.0 (Olympus) according to in-home image analysis protocol [29]. For spleen examinations, only red pulp within the tissue was taken into account during analyses. Iron content was calculated as a ratio of area and intensity of iron-positive stains (blue color) to the area of cell nuclei within an image.

### Iron Concentration in Tested Tissues Measured with Atomic Absorption Spectrometry

Previously collected, stored in −20 °C, and subsequently thawed tissue samples were prepared for further quantitative assessment of iron concentration with atomic absorption spectrometry (AAS) method, according to the protocol described in details before [24]. Briefly, tissues were weighted and kept for > 16 h in the solution contained 5 ml of 65% nitric acid and 1 ml of 30% hydrogen peroxide (Merck). Subsequently, samples were mineralized in the microwave system Ethos 900 (Milestone, USA) into liquid solution and evaluated with AAS (Perkin-Elmer) for iron content.

### Statistical Analysis

Data obtained in the study were presented as a mean ± standard error of the mean (SEM). For statistical evaluation of iron accumulation obtained with Perl’s staining, one-way ANOVA with Tukey–Kramer multiple comparison post hoc test were performed for all groups. For evaluating a significant difference between control and experimental groups from AAS analyses, an unpaired *t* test was used. Significant differences between control and experimental groups in in vitro experiments were evaluated using one-way ANOVA with Dunnett multiple comparisons test. Statistical analyses were calculated with GraphPad InStat 3.10. For all tests, obtained results were considered as statistically significant with *P* ≤ 0.05 and *P* ≤ 0.01 and *P* ≤ 0.001 as highly and extremely significant.

## Results

### Characterization of Nanoparticles

**Fig. 1:**
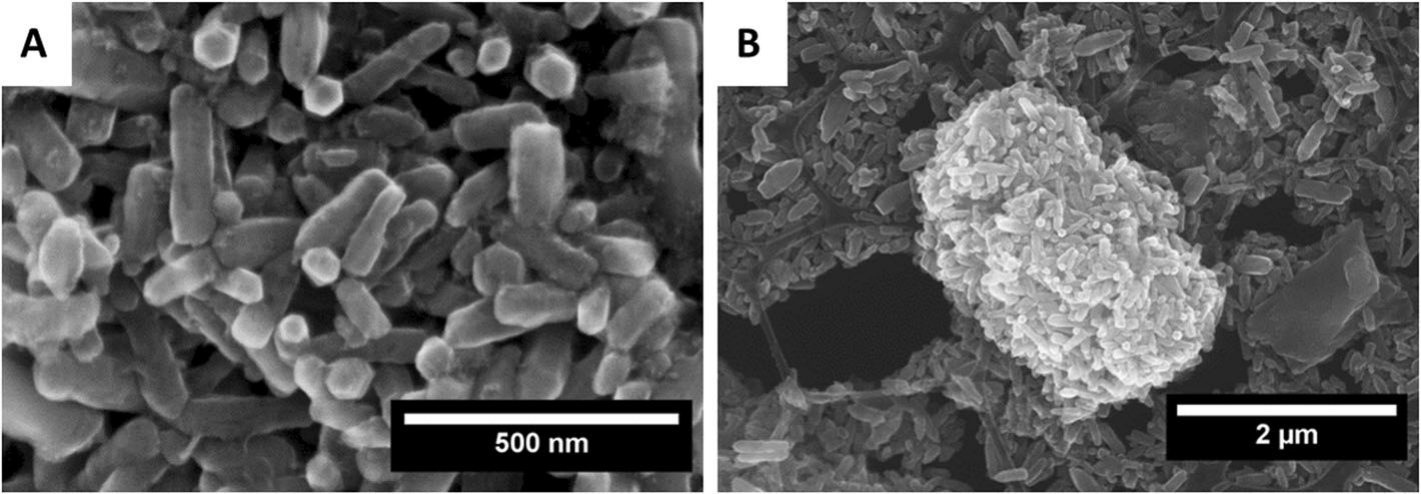
Scanning electron microscopy images of ZnO:Fe NPs deposited on copper mesh after sedimentation of large aggregates. Magnification 100 kx (**a**) and 20 kx (**b**). Suspension containing 1 mg of ZnO nanoparticles per milliliter of water was dropwise put on the polycarbonate-coated copper mesh to enable high-resolution scanning microscopy observations

Scanning electron microscopy (SEM) images of as crystallized in microwave hydrothermal process ZnO:Fe nanocrystals are shown in Fig. 1. The dry powder stored after the synthesis was dispersed in water using ultrasonic bath, and then deposited on the copper 400 mesh. Suspension resulting from the ultrasonic treatment was left for 2 h for agglomeration and sedimentation of large grains. In the sample commonly are seen ZnO crystals shaped in the form of elongated hexagonal prisms. Crystals are elongated in the direction of c-planes growth—[001]. The intersection of prisms is clearly seen on the image: hexagons are 50–100 nm in size. Basically, nanoparticles are agglomerated and form larger structures. In Fig. 1b, there is shown such structure of heavily agglomerated and grouped ZnO:Fe crystals. The sample is also composed of non-uniform grains which size distribution contains between hundreds nanometers and micrometers. There is not clear indication of separate other than ZnO phase existence, suggesting absence of iron-based phase (e.g. iron oxide) crystallization [30].

**Fig. 2:**
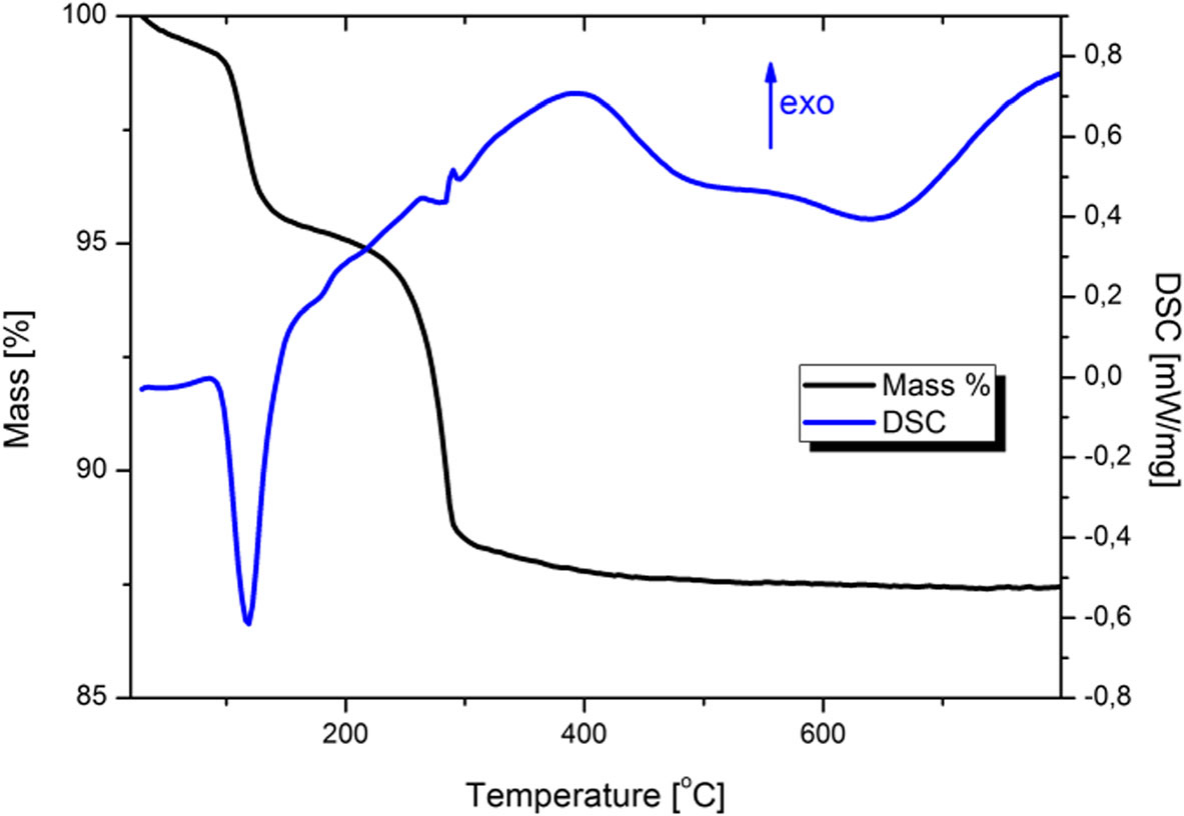
TGA/DSC of ZnO:Fe NPs heated in argon

Thermogravimetric analysis (TGA) and differential scanning calorimetry (DSC) were performed under the flow of argon from room temperature to 800 °C and the obtained results are shown in Fig. 2. The first mass loss took a place up to 200 °C and it caused a decrease of sample mass by 4.78% regarding to its initial mass. The second loss was registered between 200 and 400 °C and its value is about 7.36%. There were no other mass decreases up to 800 °C. First one was related to the evaporation of surface adsorbed water molecules and the second one was likely caused by a reduction of hydroxyl groups. DSC curve shows endothermic peak at 118.3 °C (− 0.6175 mW/mg, evaporation of water) and exothermic peak at 283.1 °C (0.4356 mW/mg, decomposition of hydroxyl groups). Small effect related to decomposition indicated that almost full crystallization of ZnO:Fe nanoparticles occurred in microwave hydrothermal process.

**Fig. 3:**
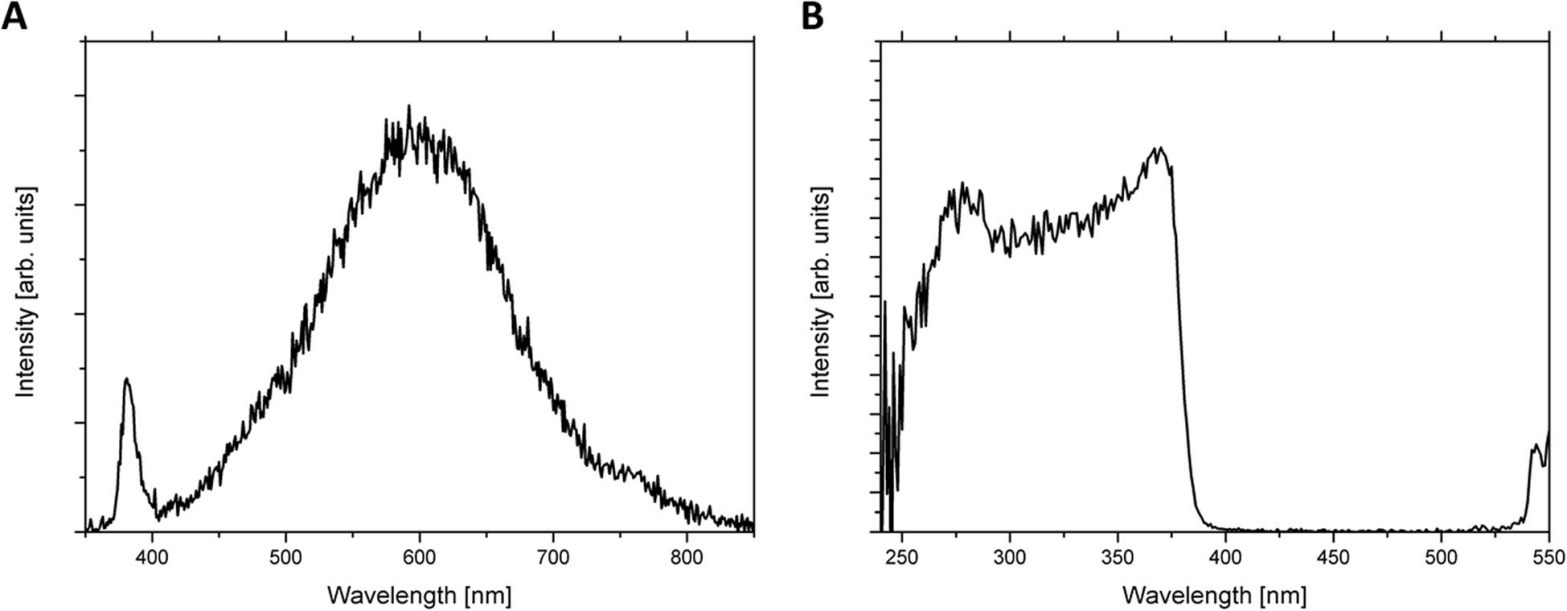
Photoluminescence (PL) emission (**a**, λ_exc_ = 260 nm) and excitation (**b**, λ_em_ = 595 nm) spectra of ZnO:Fe NPs. Samples were loosely poured into the aluminum matrix then rebalanced to enable PL measurements. Second and higher orders of excitation wavelength were filtered with spectral bandpass filter

Figure 3 shows room temperature photoluminescence spectra of ZnO:Fe NPs. Emission spectrum (Fig. 3a) consists of two features: low intensity narrow near band edge (NBE) luminescence and very intense broad deep level emission (DLE) luminescence. First one peaks at ~ 380 nm while the second one at 600 nm. Dominance of DLE intensity on the spectrum suggests that obtained nanocrystals were strongly defected on the crystallographic level [31–33]. Excitation spectrum (Fig. 3b) indicates the mechanism of emission from deep levels in the bandgap of ZnO:Fe.

**Fig. 4:**
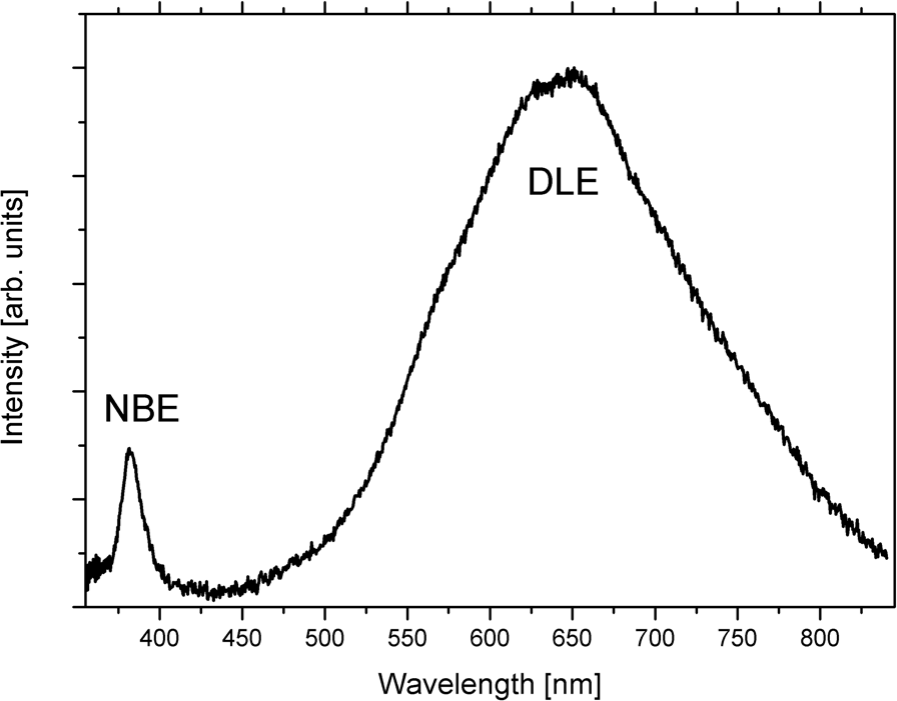
Cathodoluminescence (CL) spectrum of ZnO:Fe NPs. Dry nanopowder was pelletized to obtain CL spectrum originating from large volume of the sample. Shown bands illustrate quantity and quality of crystallographic defects present in zinc oxide nanocrystals

Cathodoluminescence spectrum of ZnO:Fe NPs is shown in Fig. 4; DLE/NBE intensity ratio is ≈ 25. NBE band peaks at ca. 380 nm, DLE band contains four components peaking at ~ 570, 625, 653 nm and very weak at 770 nm. According to McCluskey and Jokela [34], 570-nm band is related to oxygen vacancies, while others were assigned to zinc vacancies in the wurtzite-type lattice [35].

Quantity of iron in the sample was determined using two techniques: atomic absorption spectroscopy (AAS) and energy dispersive X-ray spectroscopy (EDX). In the case of AAS, the suspension prepared similarly as used in rodents experiment was analyzed to indicate 3.98 of atomic % Fe in the ZnO:Fe. By EDX method, dry powder was analyzed in the copper mesh to find concentration of iron is 4.5%at. in relation to the zinc ions.

**Fig. 5:**
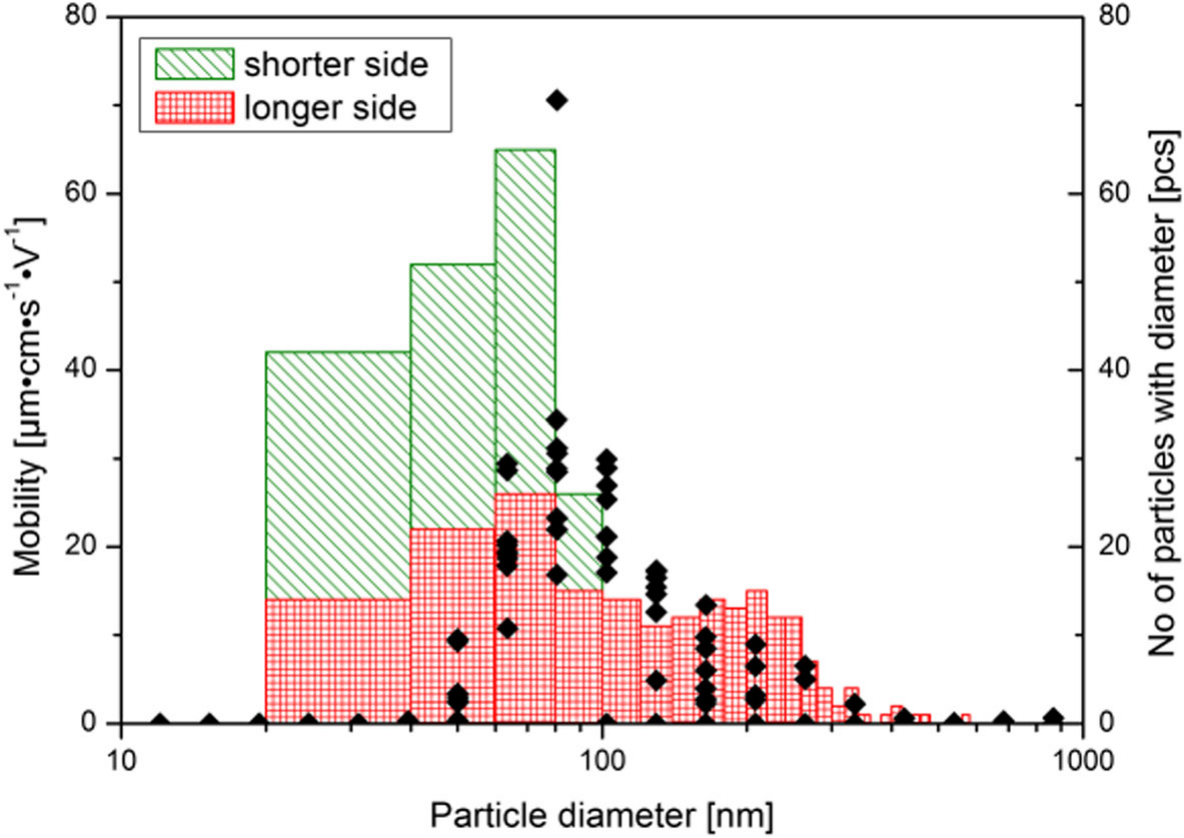
Distribution of nanoparticles’ hydrodynamic radii as measured in water suspension (dots) and taken directly from TEM images along shorter and longer sides of the nanocrystals (see text). Nanoparticles were transferred to water suspension by high power ultrasonic horn. Concentration of dry material was 1 mg per 1 ml of water. After sedimentation has finished, ten measurements of mobility were performed to obtain credible mobility vs. nanoparticle diameter plot. TEM sizes were taken directly from images: along *c* axis (longer sides of ZnO crystals) and along *m* and *a* axes (shorter sides of crystals)

The suspension properties were measured using DLS and TEM methods, with distribution of nanoparticles’ diameters shown in Fig. 5. Distribution is bimodal: for one population, most frequent diameter was peaking between 20 and 100 nm (TEM) as well as between 50 and 200 nm (DLS). The second population contains lower quantity of larger objects 100–500 nm (TEM) and ca. 150–500 nm (DLS). Bimodal character of nanoparticles is clearly observed by both shown methods. High dispersion of sizes in the larger population indicates that it corresponds to agglomerated nanoparticles. Statistics shown for diameters above 100 nm indicates many different ways ZnO:Fe nanoparticles are agglomerating. The nanoparticles in water suspension were negatively charged as revealed by zeta potential below 0 V. Value of approximately − 40 mV also indicated that the final water suspension was stable and nanoparticles did not have affinity for agglomeration.

### In Vitro Toxicological Evaluation of ZnO:Fe Nanoparticles

**Fig. 6:**
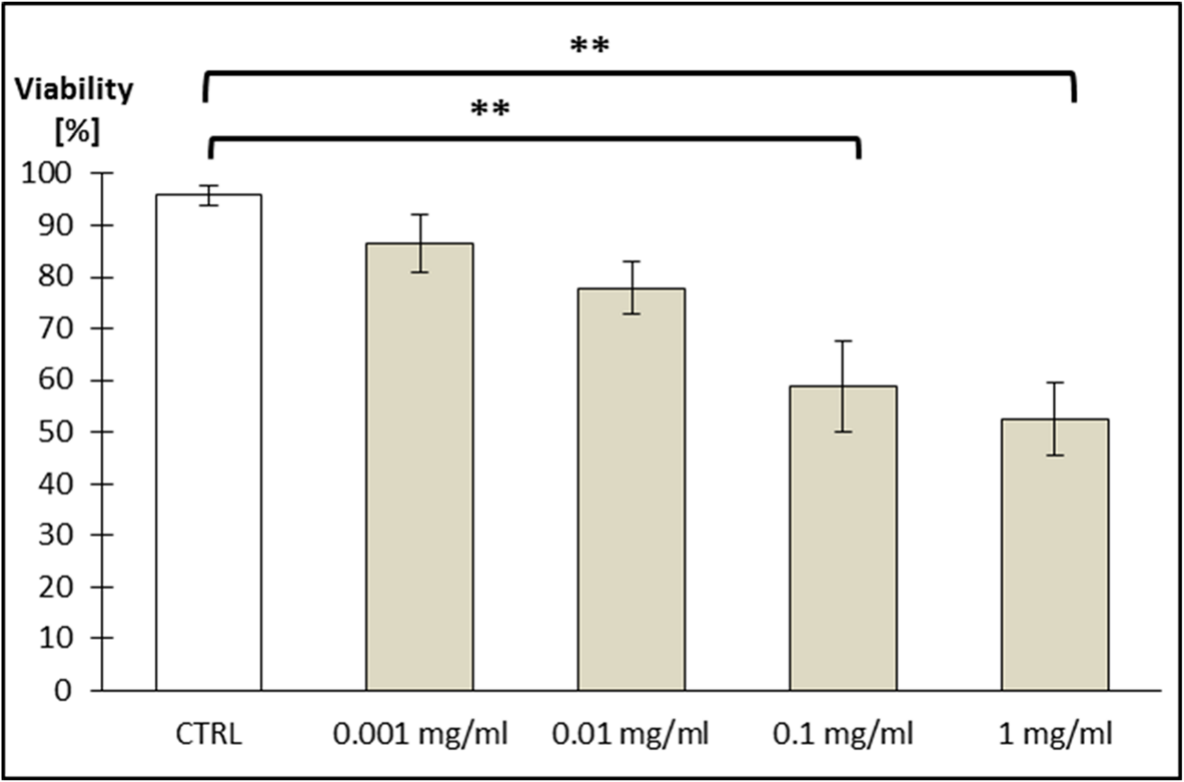
Cell viability (Caco-2 line) tested by XTT method. Cells were exposed for 24 h to a suspension of ZnO NPs doped with iron in different concentrations as follows: 0.001 mg/ml; 0.01 mg/ml; 0.1 mg/ml; and 1 mg/ml. Results represent mean percentage of viable cells (±SEM) for control and experimental groups (*n* = 4). Significant difference of **P ≤ 0.01 vs. control was marked on the graph

**Fig. 7:**
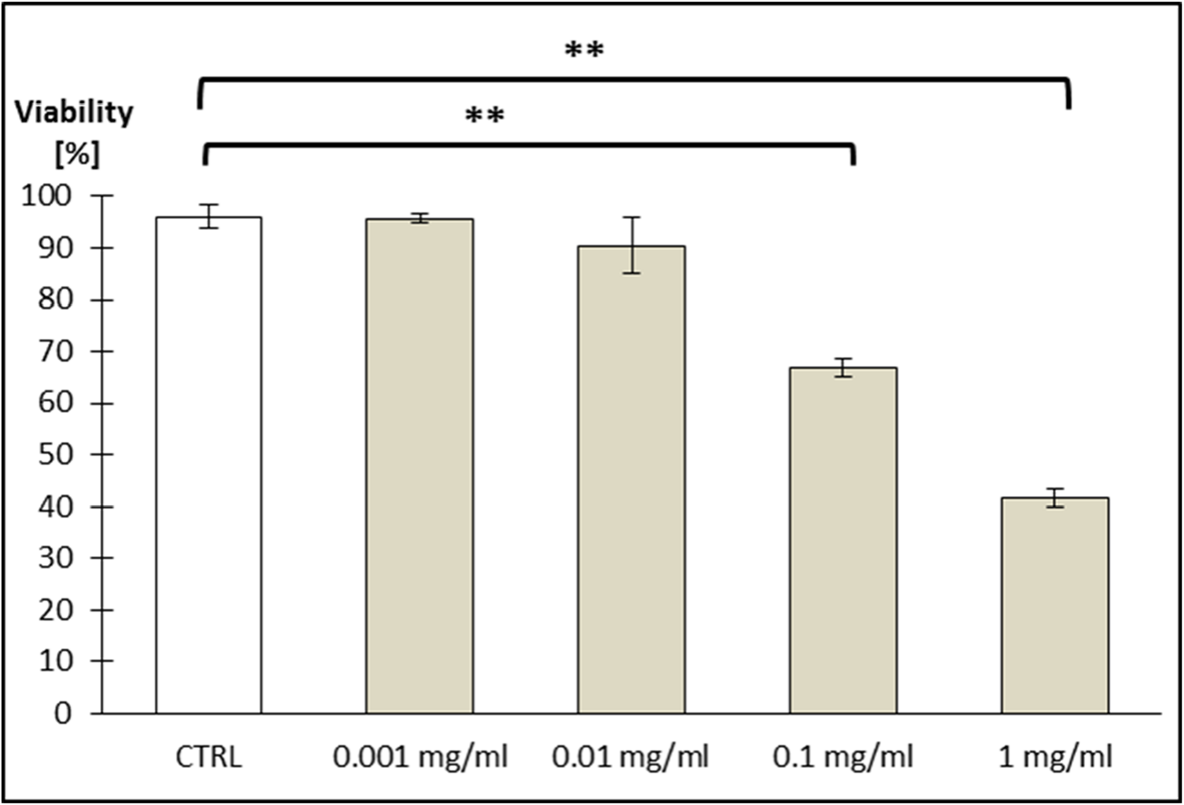
Cell viability (Caco-2 line) tested with Trypan blue staining. Cells were exposed for 24 h to a suspension of ZnO NPs doped with iron in different concentrations as follows: 0.001 mg/ml; 0.01 mg/ml; 0.1 mg/ml; and 1 mg/ml. Results represent mean percentage of viable cells (±SEM) for control and experimental groups (*n* = 3). Significant difference of **P ≤ 0.01 vs. control was marked on the graph

Both cell viability analyses (XTT and Trypan Blue) revealed decreased absorbance of Caco-2 cells incubated with ZnO:Fe NPs, which directly correlated with the number of viable cells. Statistically significant changes were observed only after 24-h exposure of incubated cells to the highest concentrations of NPs (1.0 and 0.1 mg/ml), while physiological levels of NPs concentration (0.01 and 0.001 mg/ml) had no significant effect on cell cultures (Figs. 6 and 7).

### Iron Distribution in Animal Model

**Fig. 8:**
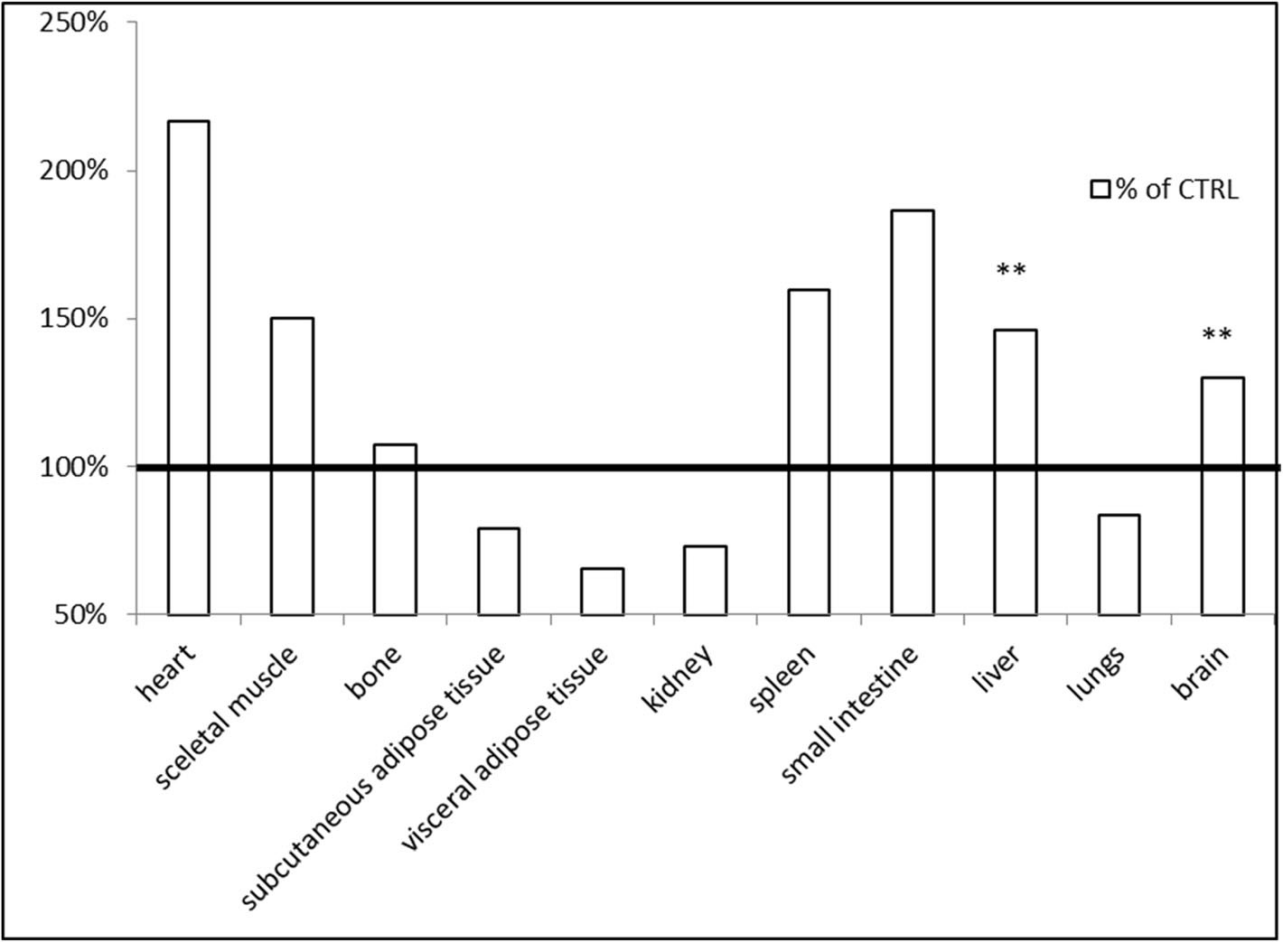
Distribution of iron within analyzed tissues 24 h following IG administration of ZnO:Fe NPs. Data represents an average percentage of Fe in tested organs from experimental group (*n =* 4) compared with the control group (100%—horizontal line). Iron content was analyzed with AAS. Significant difference of **P ≤ 0.01 vs. control

For assessing an in vivo iron accessibility from ZnO:Fe NPs, mice (*n* = 4) orally received suspension of ZnO:Fe NPs and following 24 h, animals were scarified with further collection of crucial tissues. Quantitative evaluation of iron content within examined tissues was analyzed with AAS method for all collected organs and calculated with Micro Image software (based on Perl’s staining) for liver, spleen, and brain tissues. Presented at Fig. 8, data show an increased level of iron content (vs. control) in heart, skeletal muscle, spleen, and small intestine tissues 24 h following oral administration of ZnO:Fe NPs (no statistically significant increase). In case of bone, the mean level of iron within the experimental group was only slightly higher than in the control result. Content of iron in liver and brain tissues was high significantly increased (*P* ≤ 0.01) 24 h after IG of ZnO:Fe NPs, vs. control group (Fig. 8). In the kidneys, visceral and subcutaneous adipose tissues, and lungs, Fe levels dropped 24 h after administration of ZnO:Fe NPs.

**Fig. 9:**
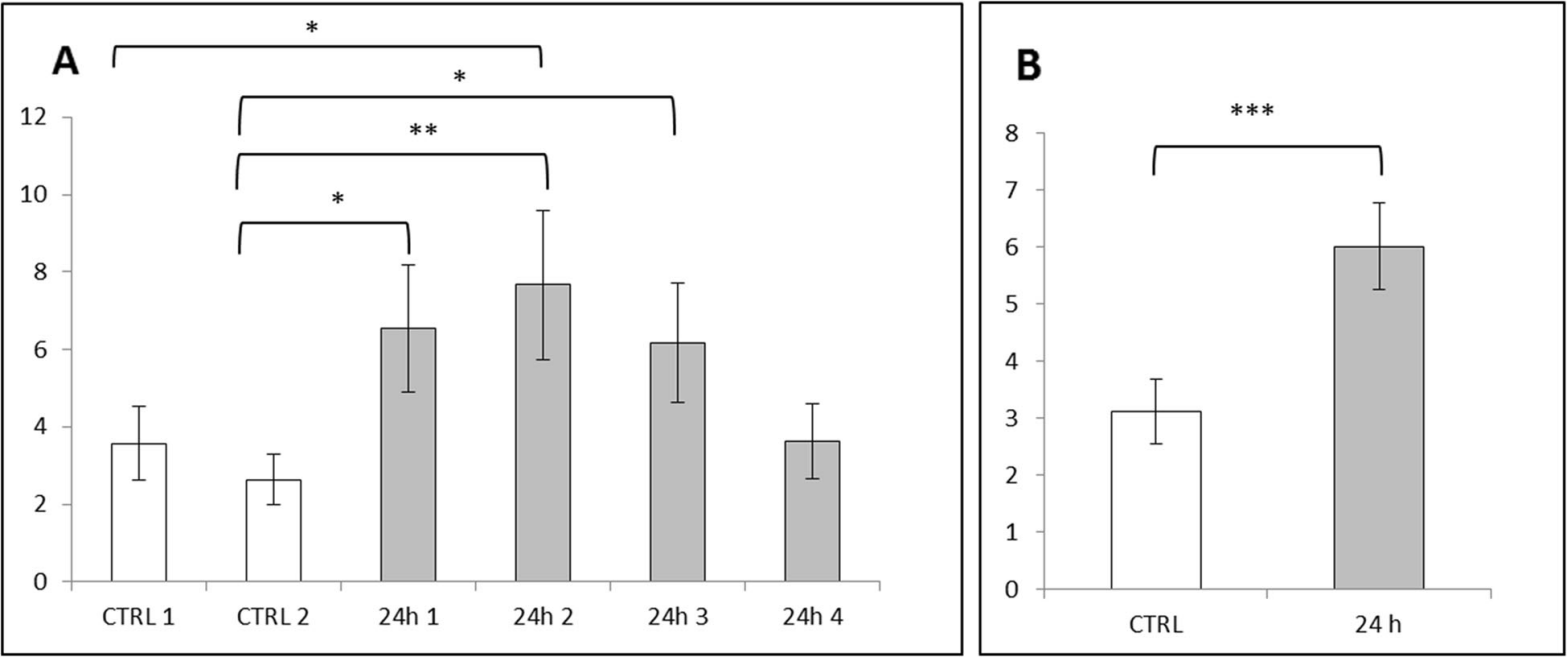
Iron content in the liver calculated with Perl’s staining and MicroImage software. Results calculated as a ratio of area and intensity of iron-positive stains to the area of cell nuclei. Data presented as mean (±SEM) result for each, examined animal (**a**) and as mean (±SEM) value for both control and experimental groups (**b**). Significant difference of *P ≤ 0.05, **P ≤ 0.01, and ***P ≤ 0.001

**Fig. 10:**
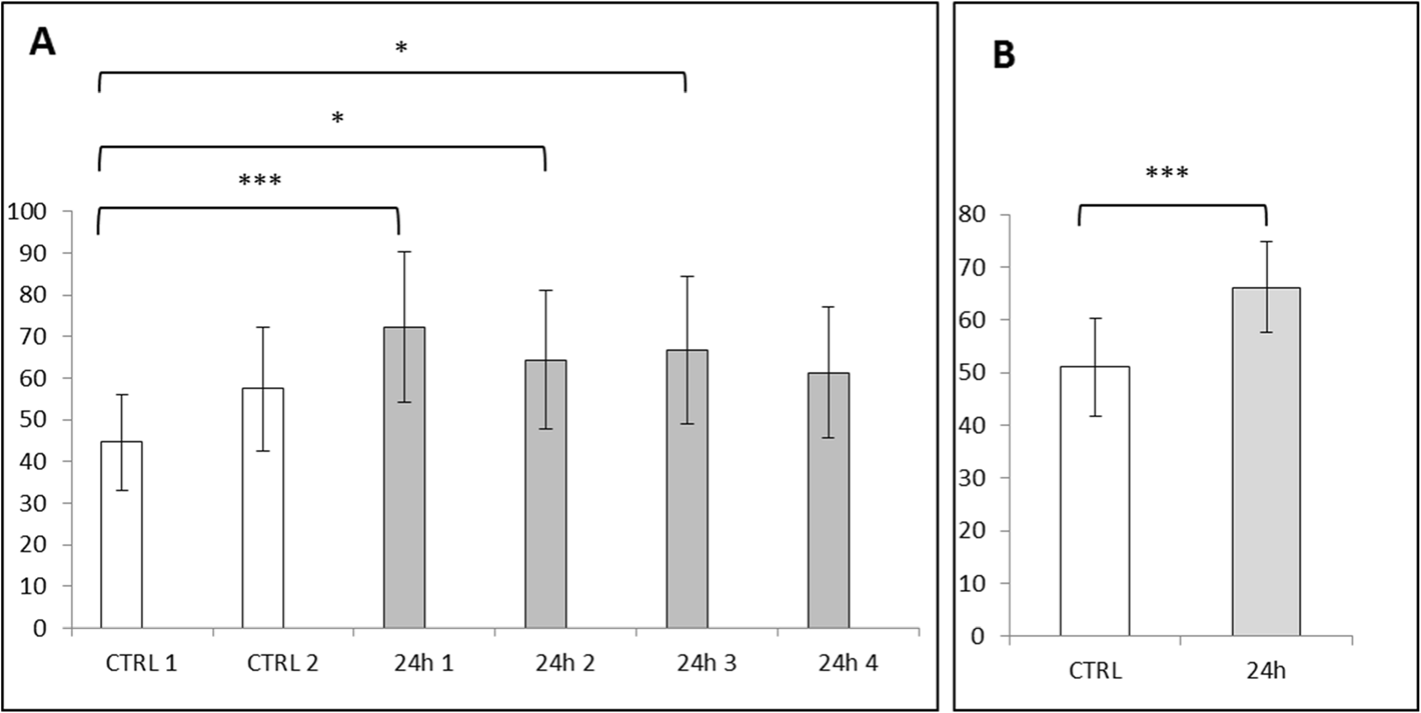
Iron content in the red pulp of the spleen calculated with Perl’s staining and MicroImage software. Results calculated as a ratio of area and intensity of iron-positive stains to the area of cell nuclei. Data presented as mean (±SEM) result for each, examined animal (**a**) and as mean (±SEM) value for both control and experimental groups (**b**). Significant difference of *P ≤ 0.05, **P ≤ 0.01, and ***P ≤ 0.001

**Fig. 11:**
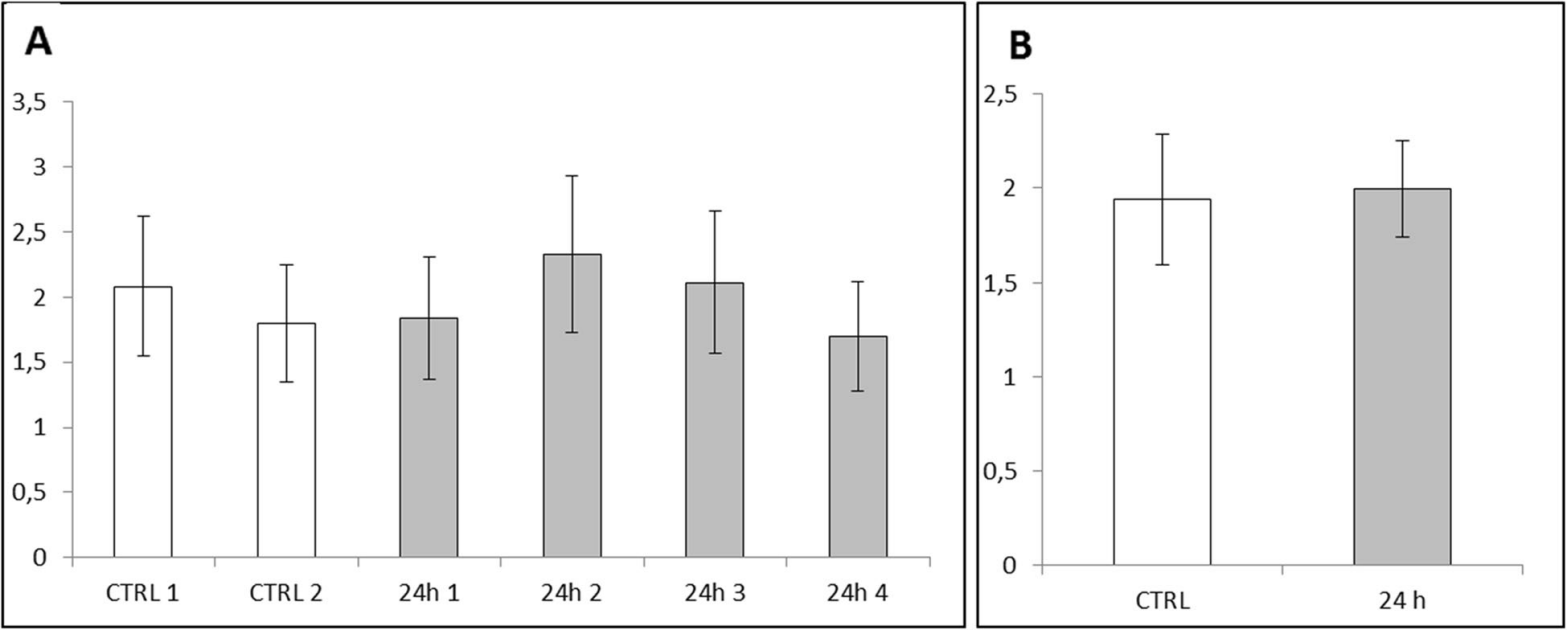
Iron content in the brain calculated with Perl’s staining and MicroImage software. Results calculated as a ratio of area and intensity of iron-positive stains to the area of cell nuclei. Data presented as mean (±SEM) result for each, examined animal (**a**) and as mean (±SEM) value for both control and experimental groups (**b**)

Evaluation of iron content stained with Perl’s method within the liver revealed an extremely significant (*P* ≤ 0.001) increased level of Fe in all experimental vs. control animals (Fig. 9b). Similarly, results obtained for the red pulp of spleen tissue showed an extremely significant (*P* ≤ 0.001) increase of iron content in all experimental vs. control animals (Fig. 10b). Data obtained from the analysis of brain tissue indicated no increase of Fe in experimental group 24 h following IG of ZnO:Fe NPs (Fig. 11b).

In case of content of iron from each animal of experimental and control groups, results were collected at the Figs. 9a, 10a, and 11a. The highest and significantly different (*P* ≤ 0.05 and *P* ≤ 0.01) level of iron within liver tissue was detected for mice – 2 (24 h 2), comparing with results from control group (Fig. 9a). Levels of iron stains in red pulps of the spleen within the experimental group were similar; however, most of them were statistically higher (*P* ≤ 0.05 or *P* ≤ 0.01) than mice – 1 (CTRL 1) from the control group (Fig. 10a). Results obtained for brain tissue were similar between each animal from experimental and control groups (Fig. 11a).

**Fig. 12:**
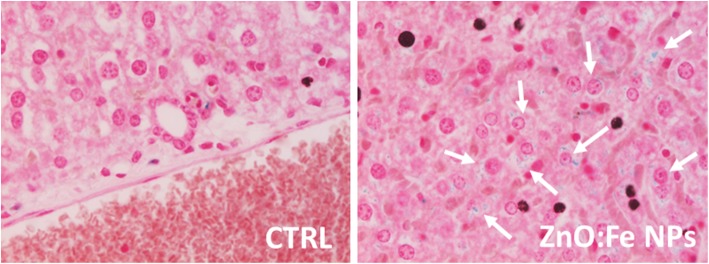
Representative microphotographs of liver sections showing staining with Perl’s method. Comparison of the presence of iron (light blue areas) in control and experimental groups of mice (with ZnO:Fe NPs administered 24 h before sacrifice). The arrows point iron deposits in hepatocytes. × 40 lens

Sections of liver and spleen tissues stained with Perl’s method were also qualitatively assessed. Increased number of iron depositions (light blue stains) was visible within hepatocytes from liver sections of experimental group (Fig. 12).

**Fig. 13:**
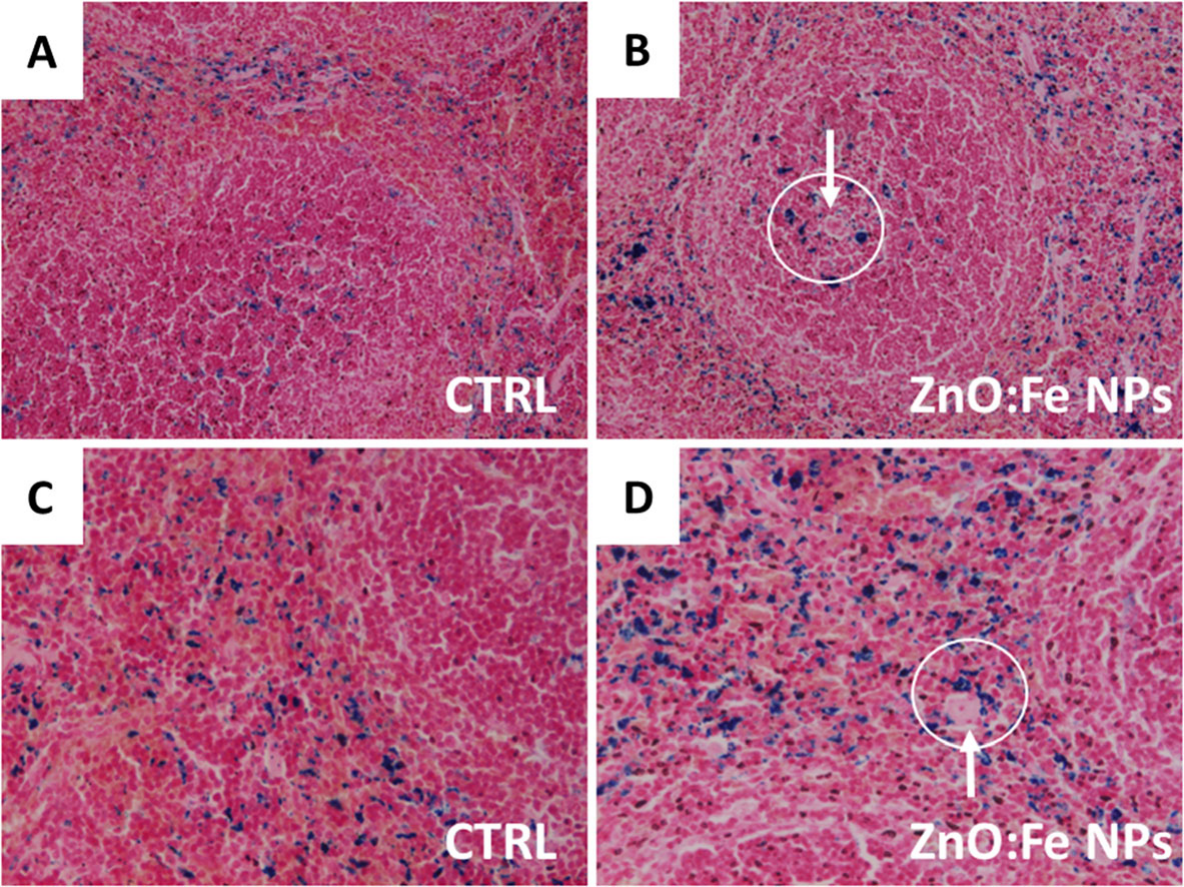
Representative spleen sections following staining with Perl’s method. Comparison of the presence of iron (blue areas ) in control and experimental group of mice (with administered 24 h before ZnO:Fe NPs). Separate presented areas of the tissue: white pulp (**a**, **b**) and red pulp (**c**, **d**). The arrows point iron accumulated around blood vessels (circles). × 10 (**a**, **b**) or × 20 (**c**, **d**) lens

General greater accumulation of iron (blue stains) within the red pulp than in white pulp of spleen was observed in both control and experimental groups (Fig. 13). After application of ZnO:Fe NPs, the higher level of iron was particularly visible within the red pulp, comparing with images from control group (Fig. 13c, d). In experimental group, an increased concentration of iron was also noticeable around blood vessels (Fig. 13b, d).

## Discussion

As was mentioned in the “Introduction” section, the iron deficiency is a considerable nutritional disorder in human population, as well as in other mammalian species [3, 7, 8]. Consequently, an implementation of new, more efficient and safe iron supplementation strategy seems to be highly desirable. Currently presented results come from preliminary studies aimed at further, detailed investigations related with potential usage of biodegradable ZnO NPs doped with Fe as a novel strategy for iron supplementation. As was confirmed previously, in-house manufactured zinc-based NPs undergo very efficient absorption after their oral administration with further, rapid distribution to major organs and tissues in the living organism [22–24]. Moreover, we reported that these nanostructures demonstrated bio-safety and biodegradability within the body along with fast clearance from the organism [36]. Employed in the current study, the oral way of NP administration provides a highly efficient way of fast delivery of the exogenous nanostructures to the body, based on persorption process [37, 38]. Furthermore, this strategy decreases chance of eventual toxic effects related with administration of iron-doped substances, because of the existence physiological “security mechanisms” related to both Zn and Fe absorption from the gastrointestinal tract. This crucial, physiological pathways of iron distribution may be avoided in case of intravenous/intramuscular application iron-doped substances [14], which can result in increased possibility of occurrence iron-related toxic side effects. The examination of the internalization pathways of the nanoparticles, their dynamics, and cellular systems involved in the process is being currently studied in the frame of other publication. In the current paper, we decided to show overall effect of our nanoparticles on living organism, focusing on the effect of iron.

Toxicity of nanostructures may be related with various features of material, not only with chemical composition, but also with e.g. size, shape, or their ability to aggregate [39]. Nevertheless, it was confirmed that smaller NPs were better absorbed than bigger one and high surface-to-volume ratio makes the particles of some metals exceptionally reactive and cytotoxic [40, 41]. Nanostructures were able to enter cells and affect their components, which altered the viability of cells [42, 43]. In one of the previous investigations on cytotoxicity of ZnO or FeO NPs, authors did not observe toxic effects (based on ability of examined cells to grow and divide), even in high doses of NPs [44]. Presented here is a study performed on Caco-2 cell line, as a model of epithelial cells of the gastrointestinal tract, revealing a low toxicity of used nanomaterials. ZnO:Fe NPs in small/physiological doses (0.001 mg/ml and 0.001 mg/ml) did not alter the viability of cells (Figs. 6 and 7). Following the incubation of Caco-2 cells with high doses of ZnO:Fe NPs, a statistically significant decrease of viability was observed. However, IC50 (the half maximal inhibitory concentration), as a measure of the effectiveness of a substance in inhibiting a specific biological or biochemical function, still remained surprisingly high—around 1 mg/ml (Figs. 6 and 7). Observed drop of cell viability was probably associated with the physical deposition of nanoparticles on the surface and overstress of the cells rather than the cytotoxic effect related to the nanoparticles themselves. In another study, authors concluded decrease IC50 of cells from cancer cell line following incubation with ZnO and ZnO:Fe3O4 NPs and relatively low cytotoxicity effect (and non-dose-depended) of examined NPs on noncancerous cells [45]. Similar observations were also reported for ZnO and platinum NPs [46, 47]. Nevertheless, further examinations on chronic toxicity profile of such composites including antioxidant activity profiling, production of reactive oxygen species (ROS), and using another cells and cell line must be performed.

Obtained in the in vivo experiment, results indicated a rapid distribution of ZnO:Fe NPs to tissues mostly related with iron homeostasis (Figs. 8, 9, and 10). Similar studies, concerning the bioavailability of iron from “nano” form, were mostly performed on anemic animals or examined following the iron depletion period [18, 19, 48], where our preliminary studies were carried out on healthy, normally maintained mice. Comparison of data presented in the current work with similar studies may be misleading because of differences in employed NPs e.g. their size, shape and compound or route, dose, and frequency of their administration to animals. Results of iron level in the liver showed significantly increased level at 24 h after application of ZnO:Fe NPs, comparing with control (Figs. 8 and 9). Iron accumulation was visible in hepatocytes of liver tissue (Fig. 12), where the major storage site of the element takes place [49]. Similarly, previous studies with intraperitoneally injected iron oxide, magnetic NPs to rats also resulted in 55% accumulation of these nanomaterials in the liver at 6 h following injection [50]. Likewise, the spleen level of iron in the current study was clearly higher in experimental group, than within control (Figs. 8 and 10). Interestingly, tendency of iron accumulation was detected around blood vessels within the spleen, which might indicate the transfer of ZnO:Fe NPs from general bloodstream into the tissue (Fig. 13b, d). Primary accumulation of nanostructures within the liver and spleen after their application might also be attributed with clearance via mononuclear phagocytes in these tissues, as was also postulated previously [50–52].

In case of brain tissue, the measurement of total iron level (AAS method) showed significantly higher level of the element in animals with previously administered ZnO:Fe NPs (Fig. 8), which was not confirmed by analysis based on Perl’s staining (Fig. 11). Inconsistency between both, employed in the study methods, may be related with sensitivity of Perl’s staining. This histological method of iron visualization is dedicated to detect iron aggregates. Iron contained in the “nano” form, additionally distributed within the tissue without physiological deposits of these elements, might not be sensitive enough to stain these extremely small deposits of iron. Possibility of overcoming the blood-brain barrier with manufactured by us nanostructures was already described [23, 24, 53]. In another study, with intraperitoneally administered superparamagnetic maghemite iron oxide NPs to mice, no accumulation of iron in experimental group was detected, nor within brain or heart tissues, which is inconsistent with our results [52]. Another study, focused on a distribution pattern of iron oxide magnetic NPs within living organism, indicated similar circulation mechanism of nanostructures as in our study—increased level of Fe within the brain and heart after administration of NPs [51]. Increased level of iron in heart tissue may be caused by presence of ZnO:Fe NPs in the general blood stream and consequently its higher level within the tissue. Likewise, higher level of iron within a skeletal muscle detected in AAS analyses is probably related with intensive blood circulation within this muscle and in case of small intestine, the improved content of iron is caused by residues of orally administered NPs.

## Conclusions

In conclusion, we performed the preliminary study on the distribution of biodegradable ZnO:Fe NPs as a perspective, new supplementation strategy in iron deficiency. Deposition of iron in the body of mice following oral administration of the nanostructures was detected within the crucial tissues, where the major storage site of the element takes place. We assumed that obtained in the study results might indicate the biodegradable ZnO:Fe NPs as a good carriers of exogenous iron in the living body. However, further research is needed to completely understand the exact mechanisms of the deposition, elimination, and influence of ZnO:Fe NPs on the body.

## Data Availability

The conclusions of the following study are based on the data presented in this manuscript.

## Abbreviations

NPs: Nanoparticles
ZnO:Fe NPs: Zinc oxide nanoparticles doped with iron
IG: Intra-gastric
ROS: Reactive oxygen species
SEM: Scanning electron microscopy
PL: Photoluminescence spectroscopy
CL: Cathodoluminescence spectroscopy
DLS: Dynamic light scattering
Caco-2 cells: Caucasian colon adenocarcinoma cells
DMEM: Dulbecco’s modification of Eagle’s medium
FBS: Fetal bovine serum
NEAA: Non-essential amino acids
PSN: Penicillin–streptomycin–neomycin
EDTA: Ethylenediaminetetraacetic acid
AAS: Atomic absorption spectrometry
SEM (in statistical analysis): Standard error of the mean
